# Supported discharge service versus inpatient care evaluation (SITE): a randomised controlled trial comparing effectiveness of an intensive community care service versus inpatient treatment as usual for adolescents with severe psychiatric disorders: self-harm, functional impairment, and educational and clinical outcomes

**DOI:** 10.1007/s00787-020-01617-1

**Published:** 2020-09-03

**Authors:** Dennis Ougrin, Richard Corrigall, Daniel Stahl, Jason Poole, Toby Zundel, Mandy Wait, Victoria Slater, Paula Reavey, Sarah Byford, John Ivens, Maarten Crommelin, Daniel Hayes, Kerry Middleton, Paul Young, Eric Taylor

**Affiliations:** King’s College London, London, UK

**Keywords:** Randomised controlled trial, Intensive community care, Inpatient, Adolescent

## Abstract

Clinical guidelines recommend intensive community care service treatment (ICCS) to reduce adolescent psychiatric inpatient care. We have previously reported that the addition of ICCS led to a substantial decrease in hospital use and improved school re-integration. The aim of this study is to undertake a randomised controlled trial (RCT) comparing an inpatient admission followed by an early discharge supported by ICCS with usual inpatient admission (treatment as usual; TAU). In this paper, we report the impact of ICCS on self-harm and other clinical and educational outcomes. 106 patients aged 12–18 admitted for psychiatric inpatient care were randomised (1:1) to either ICCS or TAU. Six months after randomisation, we compared the two treatment arms on the number and severity of self-harm episodes, the functional impairment, severity of psychiatric symptoms, clinical improvement, reading and mathematical ability, weight, height and the use of psychological therapy and medication. At six-month follow-up, there were no differences between the two groups on most measures. Patients receiving ICCS were significantly less likely to report multiple episodes (five or more) of self-harm (OR = 0.18, 95% CI: 0.05–0.64). Patients admitted to private inpatient units spent on average 118.4 (95% CI: 28.2–208.6) fewer days in hospitals if they were in the ICCS group compared to TAU. The addition of ICCS to TAU may lower the risk of multiple self-harm and may reduce the duration of inpatient stay, especially in those patients admitted for private care. Early discharge with ICCS appears to be a viable alternative to standard inpatient treatment.

## Introduction

Despite a considerable increase in the number of alternatives to inpatient admission, the absolute number of young people admitted for psychiatric inpatient care in England has remained relatively stable in the past three years. In 2018, 4703 youths were admitted (of which 1741 were admitted to private units, and the rest to National Health Service (NHS) units versus 4677 (1680 to private units) in 2017 and 4670 (1637 to private units) in 2016 [15]. However, there had been a twofold increase in the number of admissions in the preceding 15 years [26].All admissions to NHS units are state-funded and free for patients. Most admissions to private units in the UK are also state-funded. They occur when there are no available suitable beds in the NHS system. During the same period, the number of referrals for mental health treatment has continued to rise in the UK, increasing by 26% between 2013 and 2018 [5]. Most young people admitted for inpatient care report a history of at least one episode of self-harm [25]. Self-harm is one of the strongest known predictors of death by suicide in young people [7], increasing the risk of death at least tenfold. The period following an inpatient admission is the period of greatest risk for suicide, increasing the risk about eightfold [6, 10, 17]. Despite recent developments in understanding risk factors [23] and treatment [27] components, there is no firm evidence on the role of inpatient treatment in managing self-harm. Little is also known about the optimal models of care for adolescents presenting with other urgent severe psychiatric disorders.

Clinical guidelines, such as those of the National Institute for Health and Care Excellence (NICE) [16], recommend Intensive Community Care Service (ICCS) for a number of disorders. However, the evidence base for these recommendations is minimal [12], despite the utmost importance of the decisions about inpatient admissions for young people’s lives and management of healthcare finances.

### Objectives

To address this gap in the evidence base, we undertook a random allocation study of an Intensive Community Care Service (called Supported Discharge Service) versus inpatient treatment as usual (TAU). This is the second quantitative paper to result from this study, in addition to a qualitative paper and the initial pilot [20, 22]. We have previously reported that ICCS was associated with shorter duration of inpatient admissions, better school integration and a reduction in multiple self-harm episodes [19, 22]. In this paper, our objectives were further comparisons between ICCS and inpatient TAU in terms of the number and severity of self-harm episodes, functional impairment, broad psychiatric symptoms, clinical improvement, reading and mathematical ability, weight, height and the use of psychological therapy and medication 6 months after the initial randomization. We also report on the differential impact of ICCS in those patients admitted to private versus NHS inpatient care. In this paper, self-harm is defined as any self-injury or self-poisoning irrespective of the suicidal intent, as outlined by the UK’s National Institute for Health and Care Excellence [14].

## Methods

### Study design

The study design has been reported in full previously [19, 22]. Briefly, this was a randomised controlled trial (RCT) of 106 young people aged 12–18 admitted for inpatient care in a rural and an urban centre in England. Young people were eligible for recruitment unless they were already known to a team with an intensive community care capability. Following the initial assessment, the young people were randomly allocated to either ICCS or inpatient TAU and followed up 6 months after the initial randomization by researchers unaware of the treatment group allocation under an intention-to-treat basis. The mean duration of ICCS treatment was 116.32 days (SD: 70.09, 95% CI: 90.61–142.03, minimum one, median 107, maximum 274 days). TAU was delivered by inpatient services, both private and NHS, followed by a return to standard outpatient care. The mean duration of standard inpatient care was 50 days (IQR: 19–125).

### Procedures

The ICCS intervention in this study was delivered by two teams, one based in London and one in a rural area in Kent. Each team included one consultant child and adolescent psychiatrist, one administrator, two to four whole-time equivalents of Child and Adolescent Mental Health Services (CAMHS) practitioners with nursing backgrounds, and two to four whole-time equivalents of clinical support workers. ICCS aimed to reduce overall length of inpatient stay and improve the quality of care by offering intensive home treatment, hospital day care and case management to young people presenting with serious mental illness. The intensity of care was flexible, up to a maximum of daily contacts. Staff tasks included assisting young people with creating customised care plans, psychiatric care, psychological interventions, helping with school re-integration, and optimising physical health care and social support. The duration of treatment varied by individual need, and the aim was to achieve transfer back to the usual community mental health service.

TAU was delivered by inpatient services and followed by a return to standard outpatient care, delivered primarily by CAMHS, with or without an interim period of hospital day care. NHS hospital inpatient care and day care were provided according to the model developed by Corrigall and Mitchell [4] unless all inpatient beds were full, in which case patients were admitted to private inpatient services. Hospital care was delivered by multidisciplinary teams, including psychiatrists, nurses, psychologists, occupational therapists, art psychotherapists, family therapists, and social workers, and led by a consultant psychiatrist. Each inpatient service had access to a hospital school. Patients in the SDS and usual care groups had access to the full range of local NHS support services open to patients in tertiary care.

### Outcome measures

In this paper, we report the following outcomes:

#### Clinical measures

1. The Self-Harm Questionnaire (SHQ) [18]. We used five or more episodes of self-harm, in line with the DSM five definition of Non-Suicidal Self Injury, to establish the proportion of young people with multiple self-harm.
2. The Clinical Global Impression—Improvement scale (CGI-I), a brief clinician-rated scale assessing clinical improvement. This scale has been validated for a range of conditions in both psychotherapy and pharmacotherapy trials [9, 11, 21, 28].
3. The Health of the Nation Outcome Scales for Children and Adolescents (HoNOSCA), a clinician-rated tool that assesses symptom severity and function across a range of psychosocial domains [8].
4. The Columbia Impairment Scale (CIS), a patient-reported measure of function [1].
5. The total number of presentations to emergency departments with self-harm using electronic patient records.
6. Height and weight, measured in cm and kg.

#### Educational outcomes

1. Reading ability measured with Wide Range Achievement Test, 4th edition.
2. Mathematical ability measured with Wide Range Achievement Test, 4th edition.
3. Behaviour during educational testing using the Guide to the Assessment of Test Session Behaviour (GATSB).

#### Process measures

1. Barriers to discharge: key problems that prevented discharge rated by the treating clinical teams and grouped into the following categories:
  - Mental state
  - Family resources
  - Community resources
  - Educational resources
  - Housing resources
  - Safeguarding concerns
  - Other
2. The total number of presentations to emergency departments, established using electronic patients records.
3. The total number of readmissions to psychiatric hospitals, established using electronic patients records.
4. The total number of occupied bed-days whilst readmitted, established using electronic patients records.
5. The total number of sessions of psychological therapy, established using electronic patients records.
6. The proportion of young people taking psychotropic medication, established using electronic patients records.
7. The proportion of young people taking antipsychotic medication, established using electronic patients records.
8. The total number of occupied bed-days in private hospitals, established using electronic patients records.

### Statistical analysis

Data were analysed using STATA 15.0. We analysed continuous outcomes using linear regression with treatment arm and baseline values of the treatment outcome (where available) as a covariate to control for possible pre-randomisation imbalances (adjusted ANCOVA approach). Other independent variables were included depending on research questions. Cohen’s d (mean difference divided by pooled standard deviation at baseline) is presented as a standardised effect size.

Categorical outcomes were analysed using the same analysis approach using logistic regression where the pre-randomisation outcome is adjusted for where it was available. We used robust standard errors for statistical tests and confidence intervals for all parametric analyses to account for possible violations of homoscedasticity and normality assumptions [24]. We used an exact logistic regression model to compare the attendance of private hospital at follow-up (yes/no) between treatment arms controlled for baseline attendance which provides more reliable statistical inference with small samples and unbalanced datasets [13]. Welch unequal variance t-test was used for group comparisons. We used an exact logistic regression model to compare the attendance of private hospital at follow-up between treatment arms controlled for baseline duration of admission [13]. Occupied inpatient days were analysed as recorded on electronic patient record systems. Multiple self-harm was analysed by comparing proportions of the young people with multiple self-harm using logistic regression.

### Role of the funding source

The funders of the study had no role in the study design, data collection, data analysis, data interpretation, or writing of the report. The corresponding author had full access to all the data in the study and had final responsibility for the decision to submit for publication. DS was part-funded by the National Institute for Health Research (NIHR) Biomedical Research Centre at South London and Maudsley NHS Foundation Trust and King’s College London. The views expressed are those of the authors and not necessarily those of the NHS, the NIHR or the Department of Health.

## Results

**Fig. 1:**
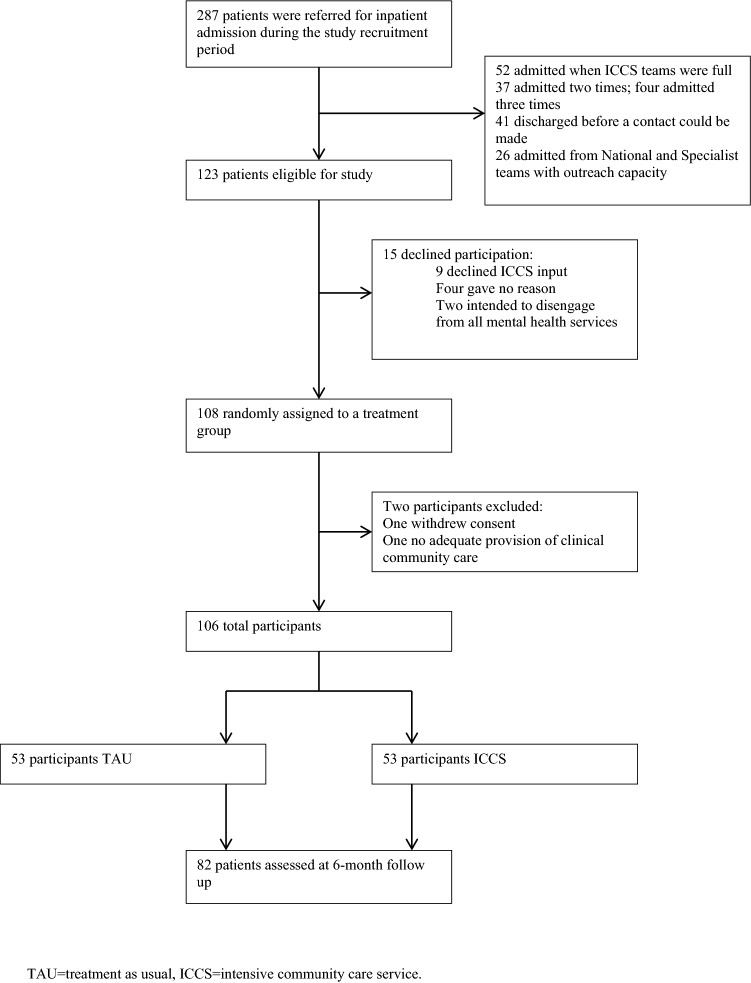
Patient flow. *TAU* treatment as usual, *ICCS* intensive community care service

287 patients were referred for inpatient admission during the study recruitment period. 123 patients were eligible for the study. 15 (12%) refused to participate. 108 patients were randomly assigned to a treatment group and 82 patients (77%) were assessed at 6-month follow-up. Electronic hospital use data were available for 100% of patients (Fig. 1).

Two patients, one in each treatment group, were withdrawn from the study. One patient, in the SDS arm, withdrew their consent and another, in the TAU arm, was withdrawn as they had no adequate provision of community clinical care and had to be looked after by an ICCS team. The final sample comprised of 106 patients.

### Sociodemographic and clinical characteristics

Data were available for all 106 patients included in the final sample, 53 in each treatment group. A descriptive comparison did not suggest major differences on any sociodemographic or clinical characteristics (Table 1).

**Table 1:** Description and comparison of demographic and clinical characteristics of treatment as usual (TAU) and intensive community care service (ICCS) groups

	TAU	ICCS
	Mean (SD) *N* = 53	Mean (SD) *N* = 53
Age (years)	16.34 (1.70)	16.23 (1.54)
Gender		
Male	20/53 (37.7)	17/53 (32.1)
Female	33/53 (62.3)	36/53 (67.9)
Ethnicity		
White British	24/53 (45.3)	28/53 (52.8)
Other	29/53 (54.7)	25/53 (47.2)
Looked after children	3 (5.6)	1 (2)
History of physical and sexual abuse	3 (5.6)	3 (5.6)
History of physical abuse	9 (17.0)	12 (22.6)
History of emotional abuse/neglect	4 (7.5)	1 (2)
History of sexual abuse	6 (11.3)	6 (11.3)
Multiple self-harm	22/49 (44.9)	32/52 (61.5)
Barriers to discharge		
Mental state	44/53 (83.0)	44/53 (83.0)
Family resources	14/53 (26.4)	15/53 (28.3)
Community resources	3/53 (5.6)	2/53 (3.7)
Educational resources	14/53 (26.4)	6/53 (11.3)
Housing resources	15/53 (13.2)	9/53 (16.9)
Safeguarding concerns	2/53 (3.8)	2/53 (3.8)
Other	1/53 (1.8)	1/53 (1.8)

#### Self-harm Questionnaire (SHQ)

SHQ data at 6-month follow-up were available for 83 patients, 45 (85%) in the ICCS arm and 38 (72%) in TAU. The proportion of patients who reported multiple (five or more) episodes of self-harm at 6-month follow-up was 16/38 (42%) in the TAU group and 11/45 (24%) in the ICCS group. Binomial logistic regression, controlling for baseline scores at pre-randomisation, revealed that adolescents randomised to ICCS were significantly less likely to report multiple episodes of self-harm, (OR = 0.18, 95% CI: 05–64, *p* = 0.008). The odds of patients in the ICCS group having multiple self-harm episodes was 82% lower than the odds of patients in the TAU group. There was no difference in the proportion of patients reporting any self-harm (OR = 1.41, 95% CI·45–4.41, *p* = 0.560).

#### Clinical global impression (CGI)

Data at 6-month follow-up were available for 87 patients, 47 (54%) in the ICCS arm and 40 (46%) in the TAU arm. Overall, CGI scores reduced from 4·27 (*n* = 104, SD = 1.17) at baseline to 3·5 (*n* = 87, SD = 1.84) at follow-up. In adjusted analyses, there were no statistically significant differences in the mean CGI score between the two groups: ICCS = 3.33 (*n* = 47, SD = 2.0), TAU = 3.70 (*n* = 40, SD = 1.62), mean difference =  − 0.50, 95% CI: − 1.12–0.19, SE = 0.35, *p* = 0.15. Binary logistic regression revealed that there was no statistically significant improvement in CGI scores (*n* = 78) at 6-month follow-up (OR 2.14, 95% CI: 0.75–6.10, *p* = 0.15).

#### Health of the nation outcome scales for children and adolescents (HoNOSCA)

HoNOSCA data at 6-month follow-up were available for 89 patients, 49 (92%) in the ICCS arm and 40 (75%) in the TAU arm. Overall, HoNOSCA scores reduced from 19·17 (*n* = 105, SD = 7.99) at baseline to 13.1 (*n* = 89, SD = 8.11) at follow-up. In adjusted analyses, there were no statistically significant differences in the mean HoNOSCA score between the two groups: ICCS = 13.25 (*n* = 49, SD = 8.1), TAU = 12·92 (*n* = 40, SD = 8.23), mean difference =  − 1.14, 95% CI: − 4·55–2.27, SE = 1.71, *p* = 0.51.

#### Columbia impairment scale (CIS)

Self-reported CIS data at 6-month follow-up were available for 83 patients, 46 (87%) in the ICCS arm and 37 (70%) in the TAU arm. Overall, CIS scores reduced from 23.27 (*n* = 105, SD = 10.91) at baseline to 18.12 (*n* = 83, SD = 10.92) at follow-up. In adjusted analyses, there were no statistically significant differences in the mean CIS score between the two groups: ICCS = 18.76 (*n* = 46, SD = 11.43), TAU = 17.32 (*n* = 37, SD = 10.36), mean difference =  − 1.83, 95% CI: − 6.10–2.44, SE = 2.15, *p* = 0.40.

#### Presentations to emergency departments with self-harm

Presentation to emergency departments with self-harm data over the 6-month follow-up were available for 106 patients, 53 (100%) in the ICCS arm and 53 (100%) in the TAU arm. Overall, the number of presentations to emergency departments with self-harm decreased from 1.35 in the 6 months preceding randomisation at baseline (*n* = 105, SD = 3.26) to 0.45 in the 6 months following randomisation (*n* = 106, SD = 1.33). In adjusted analyses, there were no statistically significant differences in the mean number of presentations to emergency departments with self-harm between the two groups: ICCS = 0.43 (*n* = 53, SD = 1.5), TAU = 0.47 (*n* = 53, SD = 1.15), mean difference = 0.14, 95% CI: − 0·31–0.59, SE = 0.23, *p* = 0.54.

#### Total presentations to emergency departments

Presentation to emergency departments data over the 6-month follow-up were available for 106 patients, 53 (100%) in the ICCS arm and 53 (100%) in the TAU arm. Overall, the number of presentations to emergency departments decreased from 3·38 (*n* = 105, SD = 5.21) in the 6 months preceding randomisation at baseline to 0.82 (*n* = 106, SD = 1.6) in the 6 months following the randomisation at follow-up. In adjusted analyses, there were no statistically significant differences in the mean presentation to emergency departments score between the two groups: ICCS = 0.83 (*n* = 53, SD = 1.67), TAU = 0.81 (*n* = 53, SD = 1.53), mean difference =  − 0.07, 95% CI: − 0.72–0.58, SE = 0·33, *p* = 0.83.

#### Readmissions to inpatient psychiatric units

Data on the proportion of patients readmitted at least one time to inpatient psychiatric units and the mean number of readmissions during the follow-up period were available for 106 patients, 53 (100%) in the ICCS arm and 53 (100%) in the TAU arm. Mean number of readmissions was 0.25 (SD = 0.51) in the TAU arm and 0.22 (SD 0.41) in the ICCS arm. Binary logistic regression revealed that there were no statistically significant differences between the groups in the proportion of the patients readmitted for any reason (OR 0.90, 95% CI: 0.35–2.27, *p* = 0.82).

#### Occupied bed-days whilst readmitted

Data regarding total number of days readmitted were available for 106 patients, 53 (100%) in the ICCS arm and 53 (100%) in the TAU arm. Overall, the total mean number of occupied bed-days whilst readmitted was 17.72 (*n* = 106, SD = 53.6) during the follow-up period. In adjusted analyses, there were no statistically significant differences in the mean number of occupied bed-days whilst readmitted between the two groups: ICCS 12.62 (*n* = 53, SD = 36.3), TAU 21.92 (*n* = 53, SD = 66.6), mean difference =  − 9.30, 95% CI: − 30.0–11.4, SE = 10.4, *p* = 0.37.

#### Hospital use in patients admitted to private versus NHS inpatient units

We had complete data on all patients for this outcome measure. At baseline, patients spent on average 40.55 days (*n* = 106, SD = 96.05) in hospitals. At baseline, 90 (84.9%) patients had NHS inpatient care only and 16 (15.1%) had at least some private inpatient care [TAU = 9 (17.0%), ICCS: 7 (13.2%)]. Ten (9.4%) patients had exclusively private care at baseline. During the 6-month follow-up period, patients with at least some private care spent 63.31 days (SD = 74.15) in hospitals, while NHS only patients spent 36.50 (SD = 99.24) days, [mean difference − 26.81 days, 95% CI − 14.72–68.36, t (106) = 1.28, *p* = 0.20]. The 16 patients with some private care at baseline spent on average 41.08 (SD = 34.38) days in private hospitals. At follow-up, 18 (16.9%) patients had at least some private care (TAU: *n* = 13, 24.5%, ICCS: *n* = 5, 9.4%) of which 14 already had private care at baseline.

An exact logistic regression model analysis revealed that patients in the ICCS group tended to be less likely to be readmitted to a private hospital than patients in TAU (OR = 0.32 (0.08–1.07, *p* = 0.07). This effect became significant after controlling for admission to a private hospital at baseline [OR: 0.10 (95% CI >  = 0.00–0.71, *p* = 0.019)].

Patients with some experience of private care at baseline spent on average 73.8 (95% CI 30.3–122.4) fewer days in private hospitals if they were in the ICCS group compared to TAU (Welch *t* test: *N* = 18, t (14.0) = 2.50, *p* = 0.025, and fewer days in any hospital, mean difference in total days in hospital: 118.4 (95% CI = 28·2–208.6, *n* = 18, Welch *t* test: t (13.9) = 2.82, *p* = 0.014).

#### Psychotropic medication

Data recording the proportion of patients taking any psychotropic medication was available for 106 patients, 53 (100%) in the ICCS arm and 53 (100%) in the TAU arm. There was no statistically significant difference in the proportion of patients taking any psychotropic medication between the treatment arms at 6-month follow-up (OR 1.24, 95% CI: 0.47–3.10, *p* = 0.64). There was also no statistically significant difference in the proportion of patients taking antipsychotic medication at follow-up (OR 0.78, 95% CI: 0.29–2.08, *p* = 0.62).

#### Weight in kg

Weight data at 6-month follow-up were available for 60 patients, 28 (47%) in the ICCS arm and 32 (53%) in the TAU arm. Overall, weight increased from 67·84 (*n* = 93, SD = 19.7) at baseline to 73.07 (*n* = 60, SD = 23.3) at follow-up. In adjusted analyses, there were no statistically significant differences in the mean weight between the two groups: ICCS = 69.78 (*n* = 28, SD = 25.6), TAU = 75.95 (*n* = 32, SD = 21.0), mean difference =  − 3.01, 95% CI: − 9.82–3.79, SE = 3.40, *p* = 0.38.

#### Height in cm

Height data at 6-month follow-up were available for 70 patients, 35 (50%) in the ICCS arm and 35 (50%) in the TAU arm. Overall, height increased from 167.55 (*n* = 97, SD = 10.0) at baseline to 168·1 (*n* = 70, SD = 9.41) at follow-up. In adjusted analyses, there were no statistically significant differences in mean height between the two groups: ICCS = 167.06 (*n* = 35, SD = 9.75), TAU = 169.13 (*n* = 35, SD = 9.07), mean difference = 0.67, 95% CI: − 0·69–2·04, SE = 0.68, *p* = 0.33.

#### Total psychological therapy sessions attended

Data on the total number of psychological therapy sessions attended over the 6-month follow-up were available for 106 patients, 53 (100%) in the ICCS arm and 53 (100%) in the TAU arm. Overall, the number of psychological therapy sessions increased from 7.76 (*n* = 106, SD = 12.4) in the 6 months preceding randomisation at baseline to 13.7 (*n* = 106, SD = 13.4) in the 6 months following randomisation. In adjusted analysis, there were no statistically significant differences in the total number of psychological therapy sessions between the two groups: ICCS 14.26 (*n* = 53, SD = 12.5), TAU 13·13 (*n* = 53, SD = 14.4), mean difference = 1.26, 95% CI: − 3.52–6.04, SE = 2.41, *p* = 0.61.

#### Reading ability

Reading ability data at 6-month follow-up were available for 49 patients, 28 (53%) in the ICCS arm and 21 (40%) in the TAU arm. Baseline reading ability data were also available for 65 patients, 34 (64%) in the ICCS arm and 31 (58%) in the TAU arm. Overall, reading ability score increased from 106·06 (*n* = 65, SD = 23.6) at baseline to 110.59 (*n* = 49, SD = 23.6) at follow-up. In adjusted analyses, there were no statistically significant differences in the mean reading ability score between the two groups: ICCS = 110.61 (*n* = 28, SD = 21.6), TAU = 110.57 (*n* = 21, SD = 26.6), mean difference =  − 2.14, 95% CI: − 14.02–9.72, SE = 5.89, *p* = 0.72.

#### Mathematical ability

Mathematical ability data at 6-month follow-up were available for 50 patients, 28 (53%) in the ICCS arm and 22 (42%) in the TAU arm. Baseline mathematical ability data were also available for 65 patients, 35 (66%) in the ICCS arm and 31 (59%) in the TAU arm. Overall, mathematical ability score increased from 91.18 (*n* = 65, SD = 15.3) at baseline to 94.16 (*n* = 50, SD = 19.0) at follow-up. In adjusted analyses, there were no statistically significant differences in the mathematical ability scores between the two groups: ICCS = 92.79 (*n* = 28, SD = 19.4), TAU = 95.91 (*n* = 22, SD = 18.6), mean difference = 0.921, 95% CI: − 4.42–6.26, SE = 2.65, *p* = 0.73.

#### Behaviour during educational testing

GATSB follow-up data were available for 35 patients, 18 (34%) in the ICCS arm and 17 (32%) in the TAU arm. GATSB baseline data were also available for 48 patients, 25 (47%) in the ICCS arm and 23 (43%) in the TAU arm. Overall, GATSB scores during educational testing decreased from 55 (*n* = 48, SD = 14.0) at baseline to 53 (*n* = 35, SD = 13.8) at follow-up. In adjusted analyses, there were no statistically significant differences in GATSB scores between the two groups: ICCS 52.67 (*n* = 18, SD = 13·8), TAU 53.35 (*n* = 17, SD = 14.2), mean difference =  − 3.88, 95% CI: − 11·5–3.69, SE = 3.71, *p* = 0.30.

#### Barriers to discharge

Data on Barriers to Discharge were available for all patients (*n* = 106) at baseline (Table 1). At the point of randomisation, in both groups, the most common barrier to discharge was the mental state of the young people (83% for both TAU and ICCS), followed by inadequate family resources to care for young people (TAU: 26.4%; ICCS: 28.33). Other important barriers to discharge included inadequate housing and inadequate educational resources in the community. There were no statistically significant differences between the treatment arms.

## Discussion

### Main findings

This is the second paper from the first UK randomised controlled trial of an Intensive Community Care Service (Supported Discharge Service, SDS) versus inpatient TAU for adolescents with severe psychiatric disorders. Confirming findings previously reported [19], young people randomised to ICCS were more than two times less likely to report multiple self-harm episodes over the 6-month follow-up. The vast majority of the occupied patient days saved with ICCS was accounted for by the young people admitted for private inpatient care. There were no statistically significant differences between the two arms on the remaining outcomes. Similar results were obtained in Germany [2, 3], by a research group independent of the authors of the SDS model developers [20].

### Comparison with other studies, meaning and implications

So far, seven trials have investigated the use of intensive community care versus inpatient treatment in children and adolescents with severe psychiatric disorders. The number of RCTs is small and very few research groups do this work [12]. A recent systematic review observed that using intensive community services is associated with clinical improvements similar to inpatient care in most studies and that, where differences in clinical outcomes existed, they tend to favour intensive community treatment.

Admitting young people to psychiatric units for longer appears to be linked with iatrogenic increase in multiple self-harm. The study found that adolescents randomised to receive ICCS, which led to shorter stays on inpatient units, were significantly less likely to report five or more episodes of self-harm at 6-month follow-up than those adolescents randomly allocated to the TAU condition. There were no differences between the two groups, however, in the proportion of young people who reported any self-harm or in those who presented to emergency departments with serious self-harm.

The findings of differential impact of ICCS in those patients admitted to private psychiatric units versus NHS psychiatric hospitals are of interest. Private psychiatric hospitals in the UK are independently managed and fee charging; whereas, NHS units are free for all. However, in reality most admissions to private units in the UK are also state-funded due to a lack of suitable bed availability in the NHS system. Generally, we found in this study that patients admitted to private psychiatric units tended to remain hospitalised longer. This could be a reflection of inefficient, profit-driven care or, alternatively, the severity of psychiatric disorders in the admitted patients. Or there could be other explanations. In any case, the findings suggest that ICCS appears more effective in reducing hospital use in patients admitted to private units versus NHS units. To the best of our knowledge, this is a novel finding.

We have found no differences between the treatment arms in any educational outcome. This is of interest as young people in the ICCS arm returned to school sooner and spent more time in education compared with TAU. This finding may be explained by the small sample size, regression to the mean or may point to a genuine absence of correlation between school attendance and academic achievement in this group of young people. Future studies should investigate this finding in more detail as, if the latter assumption is true, it might be important to re-think the way education is provided to young people with severe psychiatric disorders.

Overall, in adolescent patients with severe psychiatric disorders requiring hospital treatment, ICCS shows no differences versus inpatient TAU on most clinical outcomes at 6-month follow-up, however, it might be associated with some benefits, such as reduced risk of repeated self-harm. ICCS should be cautiously considered for implementation by other treatment centres.

### Strengths and limitations

Key strengths of the SITE trial lie in its pragmatic nature, broad inclusion criteria, replication of our findings by an independent research group in Germany, which has a different, insurance-based healthcare system and complete follow-up data on hospital-based outcomes due to using an electronic patient records system.

The study has significant limitations. The care received in the inpatient TAU arm was not standardised and it may be that the difference in multiple self-harm found was due to poor self-harm management in a handful of inpatient units. The study had a small sample size and as such may have failed to detect important differences between the treatment arms. Just under a quarter (23%) of those patients randomly assigned to a treatment group were not assessed at 6-month follow-up. ICCS models vary significantly in different locations and a uniform definition, minimum requirements and fidelity scales appropriate for young people are not currently available.

## Future research

In future research, it would be important to demonstrate cost effectiveness as well as effectiveness of ICCS beyond a six-month follow-up. It is unclear, at present, whether ICCS could act as an alternative to admission, rather than as a service that could reduce the duration of admissions. There might be important subgroups of young people that might benefit or be harmed by ICCS and larger studies are needed.
